# Bioactive Molecules from Marine Diatoms and Their Value for the Nutraceutical Industry

**DOI:** 10.3390/nu15020464

**Published:** 2023-01-16

**Authors:** Paola Nieri, Sara Carpi, Roberta Esposito, Maria Costantini, Valerio Zupo

**Affiliations:** 1 Department of Pharmacy, University of Pisa, Via Bonanno 6, 56126 Pisa, Italy; 2 Interdepartmental Center of Marine Pharmacology, University of Pisa, Via Bonanno 6, 56126 Pisa, Italy; 3 National Enterprise for NanoScience and Nanotechnology (NEST), Piazza San Silvestro, 56127 Pisa, Italy; 4 Stazione Zoologica Antorn Dohrn, Department of Ecosustainable Marine Biotechnology, Via Ammiraglio Ferdinando Acton, 80133 Naples, Italy; 5 Department of Biology, University of Naples Federico II, Complesso Universitario di Monte Sant’Angelo, Via Cinthia 21, 80126 Naples, Italy; 6 Stazione Zoologica Antorn Dohrn, Department of Ecosustainable Marine Biotechnology, Ischia Marine Centre, 80077 Ischia, Italy

**Keywords:** diatoms, nutraceuticals, polysaccharides, polyphenols, carotenoids, fatty acids, proteins, peptides, sterols, dietary supplements

## Abstract

The search for novel sources of nutrients is among the basic goals for achievement of sustainable progress. In this context, microalgae are relevant organisms, being rich in high-value compounds and able to grow in open ponds or photobioreactors, thus enabling profitable exploitation of aquatic resources. Microalgae, a huge taxon containing photosynthetic microorganisms living in freshwater, as well as in brackish and marine waters, typically unicellular and eukaryotic, include green algae (*Chlorophyceae*), red algae (*Rhodophyceae*), brown algae (*Phaeophyceae*) and diatoms (*Bacillariophyceae*). In recent decades, diatoms have been considered the most sustainable sources of nutrients for humans with respect to other microalgae. This review focuses on studies exploring their bio-pharmacological activities when relevant for human disease prevention and/or treatment. In addition, we considered diatoms and their extracts (or purified compounds) when relevant for specific nutraceutical applications.

## 1. Introduction

### 1.1. Nutraceuticals from the Sea

Nutraceuticals, as generally agreed, must provide health benefits, including facilitation of healing processes, when ingested by diseased individuals [1]. The term “nutraceutical” was suggested for the first time in 1989 by the New York Foundation for Innovation in Medicine to categorize a growing area of biomedical research [2]. Any substance that may be considered a food or a part of a food and provides medical or health benefits (including prevention and treatment of diseases) is, consequently, termed as a “nutraceutical” [3]. Nutraceuticals should also prevent diseases, and this is the reason why health-conscious citizens consider them as a major option for healthcare. Functional foods, which may be considered among the main instruments for nutraceutical activities, are foods that, beyond their classical meaning of “providing basic nutrition”, specifically promote human health. The boundary between nutraceuticals and functional foods is ambiguous: the main difference is represented by the format in which they are consumed. Nutraceuticals are ordinarily consumed in the form of capsules, pills and tablets, such as ordinary drugs, while functional foods are consumed as daily foods [4].

Actually, any food influences the consumer’s health in some way by interacting with various physiologic functions. Yet, in the ancient world, Hippocrates recognized the crucial relationship between food and health and emphasized that “*different diseases depend on nutrition*”. However, newer foods having the potential to promote health in specific ways were not anticipated by traditional nutrition science until the last century. Scientific research is aimed at identifying newer functional foods and nutraceutical elements by screening thousands of natural products, natural bioactive compounds and dietary supplements mainly extracted from land-based species as herbs, fermented materials, bacterial turfs, fruits and parts of animals [5].

Functional foods deriving from plants have gradually become very interesting for their physiologic benefits for humans, and their most abundant and widely distributed chemical components are represented by polyphenols, which naturally occur in vegetables, fruits, cereals, tea and coffee [6]. The potential role of functional foods based on plant-derived polyphenolic compounds includes prevention of such chronic diseases as diabetes, hypertension and cancer. The most important compounds are: i. curcumin, catechin, epigallocatechin gallate, silymarin and apigenin, exhibiting antioxidant activity; ii. resveratrol, baicalin, rutin, hesperetin and silybin, exhibiting anti-inflammatory activity; iii. quercetin and hypericin, with anticancer activity; iv. tea polyphenols and rutin, with antimicrobial activity; v. gallic acid and daidzein, with pro-oxidant activity. Several examples are reported in the literature on promising development and application of plant polyphenols encapsulated and delivered through nanocarriers, mainly represented by biopolymers (protein-based, polysaccharide-based and lipid-based carriers) with excellent properties, such as being biodegradable and biocompatible. Several sterols of plant origin, known as phytosterols, are extensively used for their therapeutic and nutraceutical properties because they exhibit cholesterol-lowering, anti-inflammatory and anti-diabetic activities [7].

Nevertheless, only a limited number of functional foods have yet been extracted from aquatic organisms, although it is known that they might represent an ideal source of compounds for nutraceutical purposes [7]. For this reason, marine nutraceuticals and functional food industries exhibited extraordinary growth in the last decade [1]. Marine environments host thousands of species that produce bioactive compounds, including vitamins, pigments, polyphenolic compounds [8,9], polyunsaturated fatty acids (PUFAs), polysaccharides, essential minerals, enzymes and peptides [7]. Consequently, seaweed and microalgae represent promising sources of compounds for nutraceutical applications [10,11,12].

### 1.2. Bioactive Compounds from Microalgae

Among microalgae, several species are interesting sources of anti-fungal [13], antituberculosis [14] and antioxidant activities [15], often in cooperation with bacteria. In fact, in many countries, food companies have already started to market functional foods containing microalgae and cyanobacteria [16,17]. Microalgae are a huge taxon containing photosynthetic microorganisms living in freshwater, as well as in brackish and marine waters, and they are typically unicellular and eukaryotic (except for cyanobacteria, a special group of photosynthetic bacteria). They comprise green algae (*Chlorophyceae*), red algae (*Rhodophyceae*), brown algae (*Phaeophyceae*) and diatoms (*Bacillariophyceae*).

Interesting examples of nutraceutical applications of diatoms are available in the literature [18], as in the case of marine diatom *Odontella aurita*, which was approved as a novel functional food in 2002. Further, in general, such microalgal active compounds as vitamins, fatty acids (FAs), carotenoids, phycobilins, polysaccharides and peptides can be used in food industries, as well as in nutraceutical, cosmeceutical and pharmaceutical applications [19]. However, exploitation of microalgae to obtain marketable products is still underexplored and extraction of high-value compounds from microalgae is restricted so far to a few species, such as *Arthrospira* spp. (a cyanobacterium commercially known as *Spirulina*), *Chlorella* spp. and *Dunaliella* spp. More recently, *Haematococcus pluvialis* (a dinoflagellate) and *Nitzschia laevis* (a diatom) have been exploited to produce astaxanthin and lipidic functional supplements [20]. As a matter of fact, diatoms are ubiquitous and comprise a diverse group of single-celled aquatic species, pigmented eukaryotic photosynthetic microorganisms often dominant in freshwater and marine environments, ascribed to the division Bacillariophyta. They are also among the primary sources of eicosapentaenoic acid (EPA) and docosahexaenoic acid (DHA) important in the marine food webs [21], both planktonic and benthic, and, usually, their growth rates are sufficiently high in culture conditions [22,23] according to the species. In addition, diatoms play an essential role in the biogeochemical cycles of carbon, nitrogen, phosphorus and silica [24] and are present in almost every type of water basin, including extreme environments [25].

Diatoms are diploid unicellular organisms actively extracting silica from the water. They need it to build their peculiar cell walls because, unlike most phytoplanktonic organisms, they use silica to construct their cell walls and are controlled by its availability and distribution [24]. In fact, external shells (frustules) are peculiar to *Bacillariophyceae* and, consequently, their taxonomical classification is based on cell wall symmetry, the number of perforations on their shells as well as type, position and organization of elegant perforations in the glass. They are strong competitors and significantly contribute to global primary production. Due to their high sinking rates, diatoms are important to the flux of carbon exported into the ocean interior [26]. Planktonic diatoms represent important food items for copepods and other small grazers, directing huge amounts of energy towards final consumers, such as fish and larger nektonic organisms [27]. Benthic diatoms, in turn, grow on any substratum, both hard bottoms and sands, and are key epiphytes of algae and seagrasses, often representing the first colonizers. However, they play different roles in planktonic and benthic environments [28]; consequently, in both conditions, they are often important regulators of consumers’ physiology [29]. In particular, diatoms produce a long list of wound-activated compounds, evolved for deterrent purposes [30,31], which may be involved in various physiologic and cellular processes, including cell-death and detoxification. Other compounds are constitutively produced and excreted in the surrounding environment or contained in special mucous substances used to improve adhesion to the substrate (in the case of benthic diatoms) [32] and to facilitate movements. The constitutive metabolites of diatoms include toxic substances used to deter their consumers [33], volatile organic compounds (VOCs) [34] and other info-chemicals, as well as anti-bacterial compounds exhibiting allo-chemical functions [35]. Finally, some diatoms produce compounds in cooperation with symbiotic bacteria [36], and, as normally observed in marine sponges [37], this cooperation increases the list of weapons and exploitable active compounds provided by benthic and planktonic diatoms [38].

**Figure 1:**
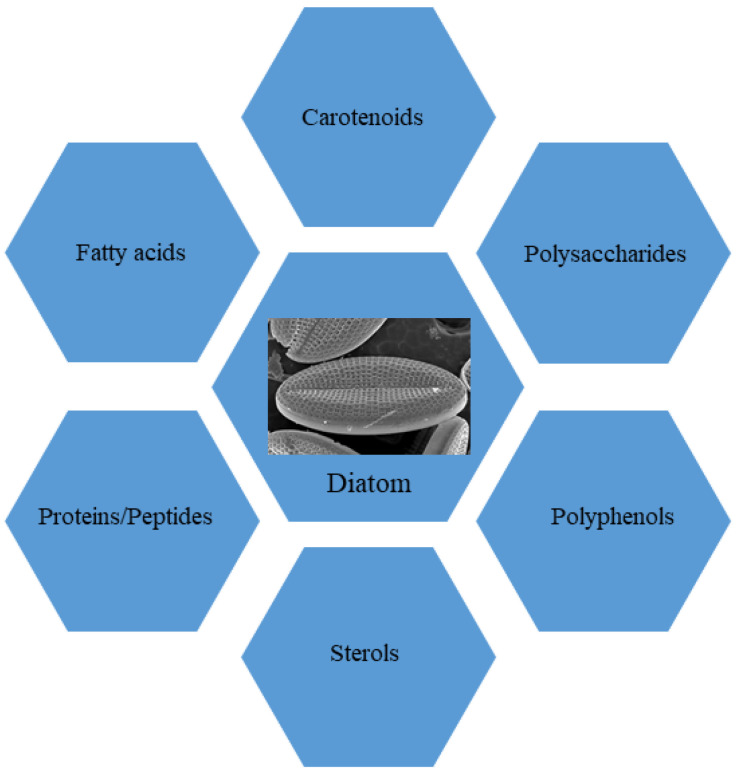
Bioactive molecules isolated from diatoms and functionally evaluated, as summarized in this review.

In this review, we focus our attention on the role of molecules isolated from marine diatoms, currently proposed as ingredients for functional foods in nutraceutical industries. In particular, we analyzed the state of the art on employment of marine diatoms in functional diets. To this aim, we conducted comprehensive searches of PubMed, analyzing the literature related to “marine diatoms and nutraceuticals” and “diatoms and dietary supplements” in order to obtain a comprehensive overview of the current knowledge on these topics. This enabled building a framework to evaluate in depth our current understanding of the selected fields. The bibliographic search revealed that diatoms represent a good source of i. primary metabolites, such as proteins, peptides, fatty acids, sterols and polysaccharides, and ii. secondary metabolites, mainly carotenoids and polyphenols (Figure 1). Marine diatoms have been considered here because investigations on freshwater diatoms are still in their infancy and only a few investigations took into consideration the nutraceutical aspects of these microalgae collected in internal basins; e.g., Sudarshan and coll. [39] investigated the anti-inflammatory properties of freshwater diatom *Nitzschia palea*. Other authors, even more recently, investigated some extracts of *N. palea* to detect in vitro and in vivo its anti-inflammatory properties using murine macrophage (RAW 264.7) cells and a mouse model [40]. However, besides a few exceptions, freshwater diatoms have been investigated mainly for their role in local communities [41] or as targets of pollutants [42] and allo-chemicals [43]. For these reasons, this review is referring to compounds produced by marine diatoms, taking into account, however, that many genera are in common with freshwater environments and several biosynthetic pathways may be present both in freshwater and marine species.

It is worth observing as well that most investigations on biotechnological applications of metabolites extracted from diatoms are based on a few species of well-studied planktonic diatoms, such as *Thalassiosira rotula*, *Chaetoceros didymus* and *Skeletonema costatum* [44], as will be demonstrated below. In fact, research on marine benthic diatoms has been primed only in the last decade by a few research groups that showed some remarkable differences in production of secondary metabolites by benthic species [24].

In contrast, most investigations on nutraceutically active compounds were performed on terrestrial plants because the interest of scientists was primed by angiosperms, easily collectible in subaerial environments, due to their antibacterial [45] or anti-cancer effects [45,46], further continued with investigation of marine organisms [27].

We also reported an overview of cultivation methods to obtain massive production of diatoms. In fact, production and storage of bioactive metabolites is largely influenced by culture techniques, which represents the greatest challenge and may help in disclosing the significant potential for future applications in the nutraceutical industry. The methods of extraction were further discussed, considering that they highly influence composition of diatom extracts to develop nutraceuticals and functional ingredients for health benefits.

## 2. Primary Metabolites

### 2.1. Proteins and Peptides

Because of their complex structure and large molecular weight, proteins share some peculiar properties, such as digestibility and absorbability in the human body less than their peptide counterparts, which, in turn, have a simpler structure and lower molecular weight [47]. Proteins provide consumers with amino acids, which are necessary for growth and development, and they also contain some peptides exhibiting positive physiological effects upon consumption [48,49], mainly used as functional ingredients in drugs for hypertension, diabetes and cancer [50]. In addition, peptides are used for other pharmacological applications due to their antimicrobial, antioxidative, anti-inflammatory, anti-amnestic and anti-thrombotic properties, as well as gastrointestinal absorption modulation, appetite suppression and immunomodulation [51]. It is important to consider that these bioactive peptides are short sequences of 2–30 amino acids joined by covalent bonds called “amide” or “peptide” bonds, released from the food matrix and maintained unaltered and easily accessible for intestinal absorption [52].

Despite their potential, bioactive peptides from diatoms are still undervalued as functional foods. *Navicula incerta* contains a high protein content (46.5–51.7%), while *Nitzschia* sp. and *Phaeodactylum* sp. are the most studied diatoms as a source of active polypeptides [53]. Two bioactive peptides, Val-Glu-Val-Leu-Pro-Pro-Ala-Glu-Leu and Pro-Gly-Trp-Asn-Gln-Trp-Phe-Leu, were isolated from *Navicula* sp. (Table 1), with potent antioxidant properties over human liver cancer HepG2/CYP2E1 cells [54].

Bioactive proteins and their hydrolysates demonstrated remarkable antioxidant properties in vitro. One aspect concerns inhibition by hydrolysates of angiotensin I-converting enzyme (ACE), involved in regulation of hypertension, acting as a catalyst for converting Angiotensin-I to Angiotensin-II, a potent vasoconstrictor peptide modulating blood pressure [55]. Therefore, exploration of microalgae strains could reveal strong and unique bioactivities, especially when these marine sources are still unexplored. Six indigenous marine diatoms, one isolate of *Bellerochea malleus*, four isolates of genus *Bellerochea* and one unidentified species of the genus *Nitzschia*, were used as sources of bioactive peptides (Table 1), showing that their protein hydrolysates have promising health benefits in management of oxidative stress and hypertension [56]. In particular, enzymatic hydrolysis was applied to protein extracts using four proteases (flavourzyme, pepsin, papain and trypsin), and the hydrolysates were then tested for angiotensin-converting enzyme (ACE)-inhibitory, antioxidant and antihypertensive properties. The results showed that papain hydrolysates derived from all microalgae strains exhibited strong ACE-inhibitory activities and antioxidant properties. In particular, protein hydrolysates from *B. malleus* were demonstrated to reduce blood pressure by 17 mmHg after 5 days of oral administration to spontaneously hypertensive rats.

Protein hydrolysates are composed of a complex mixture of free amino acids and peptides of different chain lengths, and antioxidant activities were attributed to these latter compounds through a mechanism that has not yet been elucidated [57]. Low molecular weight, the sequence of amino acids and hydrophobicity have been positively related to the antioxidant activity. However, the main characteristic is an indole/imidazole/pyrrolidine ring next to a steric structure at the *C*- and *N*-terminal region. There is evidence about the importance of the *C*-terminal region over the *N*-terminal for radical scavenging and antioxidant potency [58]. Alzahrani et al. [58] compared the antioxidant activities of protein extracts and hydrolysates (obtained by using alcalase, flavourzyme and trypsin) obtained from two microalgae (*Chlorella* spp. and *Spirulina*) with those from *N. laevis*. The antioxidant activity was enhanced by the hydrolysis process, especially when alcalase was used. In particular, the results showed that *Nitzschia* had the highest reducing capacity (2.4 ± 0.02 mg GAE/100 g) after 90 min of hydrolysis with alcalase.

**Table 1:** Proteins, peptides, fatty acids, carotenoids, polysaccharides, sterols and polyphenols isolated from several species of diatoms, along with their biological activities.

	Diatom Species	Compound	Biological Activities	References
**Proteins/Peptides**				
	*B. malleus*, *Bellerochea*, *Nitzschia*	Protein hydrolysates	ACE-inhibitory and antioxidant	[59]
	One isolate of *B. malleus*, four isolates of the genus *Bellerochea* and one unidentified species of the genus *Nitzschia*	Papain hydrolysates	ACE-inhibitory and antioxidant	[56]
	*Navicula* sp.	Val-Glu-Val-Leu-Pro-Pro-Ala-Glu-Leu; Pro-Gly-Trp-Asn-Gln-Trp-Phe-Leu	Antioxidant	[54]
**Fatty acids**				
	*P. borealis*	EPA-rich biomass	Decreasing cholesterol and triacylglycerol and antioxidant	[60]
	*Anomoenois* sp. *and Rhopalodia* sp.	Fatty acids	ACE-inhibitory and antioxidant	[61]
	*P. tricornutum*	EPA-rich biomass	Anti-aging (clinical trial)	[62]
**Carotenoids**				
	*N. laevis*	Fucoxanthinol	Anti-inflammatory, neuroprotective	[55]
	*P. tricornutum*	Fucoxanthin	Antiproliferative Antioxidant	[59]
	*P. tricornutum*	Fucoxanthin	Anti-inflammatory	[63]
**Polysaccharides**				
	*D. geminata*	Crude polysaccharides	Antioxidant and anti-inflammatory	[64]
	*Halamphora* sp.	Sulphated polysaccharide PK3	Immunostimulant (enhanced phagocytosis of macrophages)	[65]
	*H. ostrearia*	Water soluble fraction	Antiviral (HSV-1)	[66]
	*N. directa*	Naviculan	Antiviral (HSV1 and 2, IFV-A and HIV)	[67]
	*Navicula* sp.	Sulphated polysaccharides	Antioxidant	[68]
	*O. aurita*	Crysolaminarin	Antioxidant	[69]
	*P. tricornutum*	Crude polysaccharides	Anti-inflammatory and immunostimulant	[70]
	*P. tricornutum*	Chrysolaminarin-rich extract	Antioxidant and immunostimulant	[71]
	*P. tricornutum*	*P. tricornutum* chrysolaminarin-rich biomass	Positive effects on gut microbiota	[72]
	*S. acus*	Chrysolaminarin compound	Cytotoxic vs colon cancer cells	[73]
**Polyphenols**				
	*N. palea*	Phenolic extract	Antioxidant Anti-hemolytic Anti-inflammatory (in vitro and in vivo models)	[40]
	*N. laevis*	Phenolic extract	Antioxidant	[74]
	*P. tricornutum*	Gallic acid, protocatecuic acid, catechin, vanillic acid, epicatechin, syringic acid, chlorogenic acid, gentisic acid, caffeic acid, coumaric acid, ferulic acid, rutin, myricetin and quercetin	Antioxidant	[75]
	*S. marinoi*	Phenolic extract	Antioxidant Photoprotective	[76]

### 2.2. Fatty Acids

**Figure 2:**
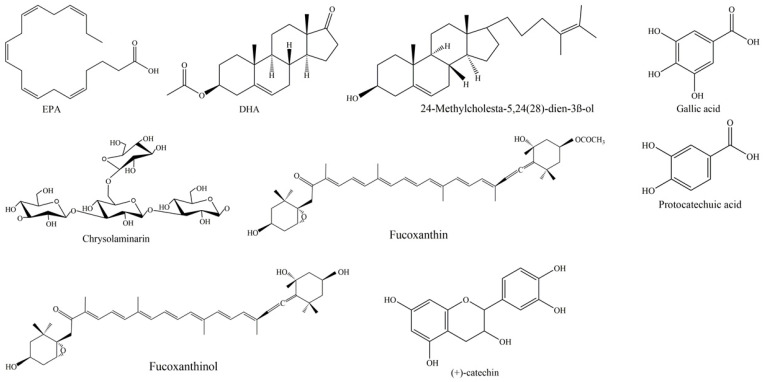
Chemical structures of the most significant molecules isolated from diatoms, drawn by using the software ChemDraw.

Fatty acids (FAs) are essential components of cell membranes, but they are also present in any organism as a means for storage of lipids. In addition, FAs are key compounds involved in signal transduction, as in the case of polyunsaturated fatty acids (PUFAs). We may define PUFAs as a category of 18–22 carbon FAs, exhibiting a straight-chain and at least two or even more double bonds. Generally, PUFAs are classified into two key families that contain FAs produced by several species through biosynthetic pathways, starting from linoleic acids (18:2*n*-2) and α-linolenic acids (18:3*n*-3), respectively. They play crucial physiologic roles and are indicated as essential FAs (EFAs) since they are produced at low efficiency by human organisms, and, consequently, their dietary introduction is needed to obtain correct presence in an organism. When they have the first double link at C6, they are indicated as *n*-6 or *ω*-6; when the first double link is located at C3, they are indicated as *n*-3 or *ω*-3. In particular, *n*-3 PUFAs contain key fatty acids, such as α-linolenic acid (ALA, 18:3*n*-3), stearidonic acid (SDA, 18:4*n*-3), eicosapentaenoic acid (EPA, 20:5*n*-3; see Figure 2 for the chemical structure) and docosahexaenoic acid (DHA, 22:6*n*-3; see Figure 2 for the chemical structure). Differently, *n*-6 PUFAs contain important FAs, such as arachidonic acid (AA, 20:4*n*-6) and γ-linoleic acid (GLA, 18:3*n*-6).

As above mentioned, various evidence indicated that *n*-3 FAs promote animal and human health and growth because they reduce inflammation processes, contribute to lower accumulation of fat in various organs and are likely to improve correct physiology of cells and tissues. In addition, EPA and DHA improve the health of the cardiovascular system and have been demonstrated to improve eye physiology and functioning of nervous system, aiding treatment of psychiatric problems. More recently, scientists have shown that a high ratio *n*-3/*n*-6 reduces risk of coronary heart disease because *ω*-6 PUFAs are to be considered less healthy than *ω*-3 due to their pro-inflammatory activity. Since *ω*-3 PUFAs are mainly contained in some fish, while they are almost rare in animal and vegetal oils, it has been stated [77] that a daily dietary dose of 250–2000 mg EPA and DHA might prevent coronary heart disease and reduce risk of fatal events. The European Food Safety Authority (EFSA) recommends a daily consumption of 250–300 mg EPA plus DHA or fish one to two times a week [78].

DHA has key functions in development of the mammalian nervous system, including development of visual systems [79], thus enhancing retinal functions and visual acuity. It plays an important role in preventing age-associated declines in cognition [80], and the decline associated with neurodegenerative diseases including Parkinson’s disease, Alzheimer’s disease and multiple sclerosis. Interestingly, EPA and DHA, as well as other PUFAs, are the nutritional constituents of tissues in many fish, crustaceans and mollusks [81], and, for this reason, it has been successfully added to several aquaculture feeds (both for adults and larvae) that were demonstrated to enhance larval growth rates and reproductive potential of adults, both in marine and freshwater vertebrates and invertebrates [82]. For all these reasons, PUFAs, particularly *ω*-3 as EPA and DHA, are needed as food and feed supplements, producing urgent demand for inexpensive DHA and EPA biological sources. Since land plants do not store EPA and DHA, the main sources of PUFAs on the market are marine organisms (both fish and invertebrates). DHA and EPA in fish oil can reach 20–30%, but the quality of fish oil can vary according to geographic regions, metal pollution, reproductive seasons, the species of fish and, eventually, off-flavors originating from lipid peroxidation [83].

However, fish and invertebrates, in many cases, are not direct producers of PUFAs through their metabolism because, in the generality of cases, FAs are accumulated through ingestion of PUFA-rich macro- and micro-algae. In recent decades, these factors led to exploitation of new resources of PUFAs by harvesting cultivated marine algae. In particular, diatoms produce and accumulate, both in the cell and outside the frustules (as extracellular polymeric substances), a range of very important bioactive metabolites, including PUFAs [84]. The content of PUFAs in some species reaches 5–6% of their dry weight and, keeping in mind their high productivity and fast turnover in bioreactors, this facilitates several biotechnological applications. An additional advantage of using diatoms instead of fish meal is the absence of cholesterol, pollutants (in bioreactor-controlled conditions), heavy metals and unwanted fishy smells.

In addition, their metabolism may be engineered and manipulated by modifying the culture conditions to improve biosynthesis of PUFAs [85]. For example, in centric diatom *Cyclotella meneghiniana*, light intensity influences production of total fatty acids (TFAs), saturated fatty acids (SFAs) and monounsaturated fatty acids (MUFAs), both at low P concentrations and high P concentrations [86]. Other studies by Hill et al. [87] indicated that the proportion of PUFAs decreases with increasing light intensity (under high phosphorus concentration, a significant increase in DHA was observed) while it increases according to the phosphorus concentration, but SFAs and MUFAs may show an opposite trend. In addition, a decline in ALA under high light intensity was accompanied by an increase in linoleic acid (LA) under increasing light. In contrast, arachidonic acid (AA) concentration was affected by light and phosphorus concentrations. Another factor drastically influencing production of FAs is temperature. For example, a decreasing production of SFA and MUFAs and an increasing production of FAs with a higher degree of unsaturation was detected in *Nitzschia frustulum* (Kützing) at lower temperatures [88], while, in *C. meneghiniana*, TFA, SFA and MUFA concentrations increased at higher temperatures and higher P supplies [86]. As a general rule, it is important to consider that slow growth of microalgae leads to a high proportion of lipids in their cells. In contrast, higher growth rates promote higher fractions of structural lipids, and PUFAs are parts of structural biomolecules, being essential components of cell membranes. Consequently, microalgae tend to accumulate neutral lipids (rich in SFAs (14:0, 16:0) and MUFAs (16:1)) at low growth rates to store excess carbon accumulated through photosynthetic processes. The demand for structural components increases at higher growth rates, leading to increasing proportions of PUFAs [89]. Finally, the plasticity of metabolic pathways of diatoms should be considered, as well as the possibility to apply genetically engineering techniques, providing an effective approach to improve PUFA production by marine diatoms.

These techniques may be adapted to selectively improve production of some classes of FAs, keeping in mind the pathways of PUFA production in diatoms [90]. The biosynthetic pathway, in fact, starts from palmitic acid (C16:0), which is transformed in stearic acid (C18:0) and further in oleic acid (C18:1), which generates LA, from which the two classical FA families spin, i.e., the *n*-6 family (GLA, Di-Homo GLA, AA, adrenic acid, tetracosatetraenoic acid, tetracosapentaenoic acid, docosapentaenoic acid) and the *n*-3 family (ALA, stearidonic acid (SDA), eicosatetraenoic acid (ETA), EPA and DHA). An alternative pathway, flowing from oleic acid through stearic acid, leads to alternative FA synthesis, leading to AA, behenic acid and lignoceric acid. This synthetic view of diatom biosynthetic strategies [69] is sufficient to show how rich and biotechnologically worthy production of essential FAs is from these microalgae.

However, it is important to keep in mind that, besides their metabolic roles as dietetic ingredients indispensable for development and metabolism of various organs and tissues, FAs may have important roles in several metabolic and regulative functions in various species. For example, in shrimp *Hippolyte inermis*, some lipophilic compounds, including a 21-C fatty acid [91], were demonstrated to promote a remarkable mechanism of sex reversal [92]. This mechanism is mediated by a cell death process that may also be applied to kill human cancer cells [37]. In parallel, the regulative functions of essential FAs (in particular the ratio *ω*-6: *ω*-3) were suggested to be important to control the inflammatory processes [93] and metabolism of sirtulins, along with vitamin D3 [94]. In particular, resolvins and protectins, as derivatives of *ω*-3, demonstrated pro-resolving anti-inflammatory activity. These arguments, along with the possibility to use diatoms as modulable sources of given classes of FAs, show their biotechnological importance and future role in production of nutraceuticals.

In this view, diatom *Odontella aurita*, rich in EPA, already received a positive opinion from EFSA regarding its use as a food ingredient and was authorized on the basis of its substantial equivalence with other authorized edible seaweeds under regulation CE n° 258/97 (https://eur-lex.europa.eu/legal-content/EN/TXT/PDF/?uri=CELEX:32017R2470&rid=1 (accessed on 5 December 2022)).

Diatom *Pinnularia borealis* accumulates high levels of EPA. For this reason, it was studied by Swiderska-Kolacz et al. [60] to test whether 7-day diet supplementation (at 1%, 3% and 5%) with this diatom could ameliorate antioxidant defense and risk of cardiovascular disease in mice. The highest concentration (5%) was shown to have protective activities, reducing the cholesterol and triacylglycerol hepatic and renal levels, while antioxidant activity, measured through glutathione content and the activity of the glutathione redox system in the same organs, was evident even at the lowest concentration. Very recently, study of FAs extracted from diatoms *Anomoenois* sp. and *Rhopalodia* sp. revealed their antioxidant activity and inhibition of angiotensin-converting enzyme (ACE), suggesting diatom lipids as alternative ACE inhibitors or food supplement for cardiovascular disease prevention due to the role played by this enzyme in hypertension and other cardiovascular disorders [61]. Another recent paper by Stiefvatter and collaborators [62] describes a pilot trial on 19 healthy elderly individuals (12 females and 7 males) receiving supplementation with EPA-rich microalga *Phaeodactylum tricornutum* for two weeks. Analyzing anti-inflammatory and antioxidative parameters, this study suggested potential useful activity of the diatom for pursuing healthy aging.

### 2.3. Sterols

Sterols (StS), a class of triterpenoid molecules, represent key components of eukaryotic life, constituting blocks of cellular membranes with action on their stability and fluidity [95]. Beneficial effects of microalgal-derived phytosterols were reported in the literature, such as anti-cancer, anti-inflammatory, antioxidant or anti-cholesteroligenic properties [96,97,98]. Sterols are frequently used as markers for diatom presence and abundance. More than 100 diatom species are able to produce over 25 different sterols, containing 27, 28 and 29 carbon atoms [7]. Many sterols are present in free forms in diatoms, even if they can also exist as conjugates, wherein the hydroxyl group in ring “A” is covalently bound to fatty acids (esterified) or sugars (glycosylated). Jaramillo-Madrid et al. [7] reviewed the occurrence of sterols in diatom species and their biological activities, such as: brassicasterol, useful in atherosclerosis prevention and hypocholesterolemia; campesterol, protective in cancer models and hypocholesterolemia; fucosterol and isofucosterol, protective in diabetes models, as well as anti-oxidant and anti-inflammatory agents; sitosterol, with activity in models of immune organisms against hypocholesterolemia.

Rampen et al. [99] reported 24-methylcholesta-5,24(28)-dien-3β-ol as the most common sterol in diatoms (see Figure 2 for the chemical structure), present in 67% of the cultures analyzed, followed by Δ5 sterols cholest-5-en-3β-ol (cholesterol), 24-methylcholest-5-en-3β-ol and 24-ethylcholest-5-en-3β-ol. Pennate diatoms preferentially contain 24-methylcholesta-5,22E-dien-3β-ol; as well, *Thalassiosirales* typically contain high relative abundances of 24-methylcholesta-5,24(28)-dien-3β-ol; *Cymatosirales* contain high relative abundances of cholesta-5,22E-dien-3β-ol; *Amphora*, *Amphiprora* and *Entomoneis* species produce 24-ethylcholesta-5,22E-dien-3β-ol; *Attheya* species contain 24-ethylcholest-5-en-3β-ol.

Enzymes 3-hydroxy-3-methyl glutaryl CoA reductase (HMGR) and squalene epoxidase (SQE) are involved in sterol biosynthesis in eukaryotes, but regulation on triterpenoid production in diatoms is not known. Overexpression of HMGR in diatom *P. tricornutum* resulted in significant differential accumulation of squalene, cycloartenol and obtusifoliol, while cycloartenol and obtusifoliol were accumulated in response to heterologous SQE overexpression [100]. Gallo et al. [101] carried out a study on biosynthesis of sterols in bloom-forming species *Skeletonema marinoi* and *Cyclotella cryptica*. In both species, sterols derive from mevalonate by cyclization of squalene to cycloartenol through cycloartenol synthase. Sterol sulfates (StS) were identified in diatom *S.a marinoi* as regulatory molecules of cell death, with a general role in diatom physiology and chemical signals in aquatic systems [102]. An efficient ultra-performance liquid chromatography–mass spectrometry (UPLC–MS) method permitted qualitative and quantitative analysis of StS from crude extract of 13 different strains of diatoms, demonstrating a species-specific distribution of StS and identifying the sulfated derivatives of 24-methylene cholesterol and 24-methyl cholesterol as the most common members in diatoms.

### 2.4. Polysaccharides

Carbohydrates are produced both in chloroplast and in the cytosol of microalgae and may be found in intracellular vacuoles stored as energy reserve compounds or inside the cell wall as structural components. In the cell wall of diatoms, polysaccharides are present with other polymers (proteins and long-chain polyamines) to coat the layer of amorphous silica over the frustules [103]. As for other microalgae, polysaccharides produced in diatoms and belonging to the class of sulfated polysaccharides (SPs) are used both to form cell wall and to be secreted in the extracellular space (extracellular polysaccharides, EPS). The primary function of the exopolysaccharide matrix is providing anchoring to a solid substrate, but also other functions may be relevant, such as motility and defense [104]. Microalgal polysaccharides have many recognized biological activities, recently reviewed by Tounsi et al. [105] and Severo et al. [106].

As far as diatoms, a biotechnologically interesting polysaccharide is chrysolaminarin (Figure 1), also called leucosin, which is bio-synthesized starting from glucose-6-phosphate (G6P). It represents the most abundant energy reserve polysaccharide in these marine microorganisms [107]. This compound, a polymer of β-1,3 glucan [103], is present in high percentages of dry weight in some diatoms, such as *O. aurita* (up to 63.11%), *P. tricornutum* (up to 25.60%) and *Thallasiosira pseudonana* (up to 23.40%) [107]. Functional investigations aimed at studying the chrysolaminarin biological activity useful for biotechnological application of diatoms were described (see Figure 2 for the chemical structure; Table 1). This polysaccharide, after isolation from *O. aurita*, was investigated by Xia and collaborators [69], who observed potent antioxidant potential. A chrysolaminarin-rich extract from well characterized diatom *P. tricornutum* [108] was studied by Carballo et al. [71] for aquaculture applications. They demonstrated high antioxidant power in vitro, and immunostimulant ability in vivo using a flatfish, the Senegalese sole, as a model. Some toxicity was also revealed by the same authors [71] both in vitro on human fibroblast cells at concentration higher than 0.001% and in vivo (1 mg/fish i.p.) using coconut oil as solvent. Unexpectedly, this dose induced 29.4% mortality, probably to be reconducted to prolonged release of chrysolaminarin from the solvent, inducing a “*sustained and potent inflammatory response*”, as authors hypothesized. A chrysolaminaran compound was also isolated from diatom *Synedra acus* [73], showing cytotoxicity against two different colon cancer cell lines with IC_50_ near 50 µg/mL. Nevertheless, the specific selectivity of this activity was not indicated. In addition, a *P. tricornutum* biomass rich in chrysolaminarin, at 5%, 15% and 25%, used as diet ingredient for adult female mice for 14 days, was evaluated for its possible beneficial effects on gut health in a preclinical setting by Stiefvatter et al. [72]; the authors observed an increase in short-chain FAs (SCFAs) at intestinal levels and a correlated positive effect on gut microbiota.

In addition to the intracellular vacuolar polysaccharides, sulfated polysaccharides SPs, well represented in the microalgal cell wall and in the extracellular matrix, received great attention for biotechnological uses. Indeed, SPs are the most studied polysaccharides from microalgae because the sulfate group confers many biological activities, such as antiviral, antibacterial, antioxidant and immunomodulatory ones [109,110,111,112]. Nevertheless, studies regarding the activity of SPs from diatoms are scarce. Three types of sulfated polysaccharides (PK1-PK3) extracted from the biomass of the photosynthetic pennate marine diatom *Halamphora* sp. were particularly abundant when Na_2_CO_3_ was used as culture supplement [65]. Among them, the PK3, rich in fucose and uronic acid, showed to be the most active, able to enhance phagocytosis by murine macrophages. This activity may be considered part of a relevant mechanism in immunomodulation-induced by SPs.

As far as the antiviral activity, it was firstly reported in 1999 for a water-soluble fraction from the marine diatom *Haslea ostrearia* inhibiting the in vitro replication of HSV-1 in “Vero” cells and delaying syncytia formation by the virus in MT4 cells [66]. More recently, the presence of a polysaccharide with antiviral activity was demonstrated from diatom *Navicula directa* by Lee et al. [67]. They isolated naviculan, a novel antiviral SP, with likely 220 kDa weight, consisting of fucose, xylose, galactose, mannose, rhamnose and other trace amounts of sugar moieties. Naviculan showed a broad antiviral spectrum against enveloped viruses, such as HSV1 and 2 (*Herpes symplex* 1 and 2), IFV-A (*Influenza A virus*) and HIV (*Human immunodeficiency virus*). This is not surprising since these viruses provide infections, with a first step represented by binding to carbohydrates on the host cell surface that may be inhibited by microalgal polysaccharides, thus hindering viral contact and penetration in cells. SPs, containing glucose, galactose, rhamnose, xylose and mannose as the main neutral sugars, were obtained by Fimbres-Olivarria et al. [68] from another *Navicula* species (*Navicula* sp.). They reported good antioxidant activity, evaluated by means of DPPH (2,2-diphenyl-1-picrylhydrazyl) and ABTS (2,2′-azino-bis(3-ethylbenzothiazoline-6-sulfonic acid) assays. Stronger antioxidant activity was observed when the diatoms were cultured under white light in comparison with red or blue radiations.

Further effects described for microalgal SPs are anti-inflammatory and immunostimulatory ones [109]. An anti-inflammatory effect was first demonstrated from diatoms using a hydrosoluble extract from *P. tricornutum*. In addition, the same extract revealed analgesic and antioxidant activities [113]. Later, polysaccharides obtained from *P. tricornutum* [70] were investigated, detecting anti-inflammatory protection active against the carrageenan-induced paw edema. The activity was greater than that of indomethacin, used as a standard drug. For the biological assays, 8-week-old females of Sprague-Dawley rats or 10-week-old females of C57BI/6 or BALB/c mice were used. Moreover, the authors showed an immunostimulatory effect by these compounds in vivo in the delayed hypersensitivity test and in vitro in the assay for phagocytic activity.

Another diatom from which the polysaccharides were isolated and functionally evaluated was *Didymosphenia geminata* [64]. The investigation addressed, for the first time, the chemistry and functional activity of crude and acidic polysaccharides, galactose and xylose being the main monomer components. Evaluation of the antioxidant activity, by means of quenching experiments of ABTS and Trolox equivalent antioxidant capacity (TEAC), as well as the influence on the expression of interleukin-6 (IL-6) and tumor necrosis factor alpha (TNF-α) in murine macrophage cell lines, revealed crude polysaccharides to be more active than acidic polysaccharides.

## 3. Secondary Metabolites

### 3.1. Carotenoids

Carotenoids are pigment molecules and, along with chlorophylls, are the most abundant pigments in diatoms. They are liposoluble terpenoids, absorbing UV with a maximum at 470 nm. They are subdivided, in nature, into two types: oxygenated carotenoids, called xanthophylls, and hydrocarbon carotenoids, simply called carotenoids. Seven types of carotenoids have been described in diatoms, i.e., fucoxanthin, diadinoxanthin, diatoxanthin, β-carotene, antheraxanthin, zeaxanthin and violaxanthin [114]. Recently, the possible interest towards diatoms as biotechnological sources of carotenoid supplements, particularly fucoxanthin (see Figure 2 for the chemical structure), has increased. Diatoms represent, in fact, a valid alternative for fucoxanthin production with respect to brown seaweeds [115]: they are more easily cultivated and a larger dry weight (DW) content may be obtained as compared to seaweed (up to 40 mg/g DW) [116]. The fucoxanthin content of diatoms is reported to be under the influence of several factors, and optimization of such parameters as pH, temperature, light, saline and aeration may be relevant for the biorefinery in this field [116]. Moreover, various extraction procedures or solvents may determine variations in fucoxanthin yields from microalgae [116].

Fucoxanthin, extracted for the first time in 1914 by R. Willstatter and H. J. Page [117] from three marine brown seaweeds, *Fucus*, *Dictyota* and *Laminaria*, has a unique chemical structure among carotenoids, with an allenic bond, and has already revealed many effects on human health. In particular, the following important biological and disease-preventive activities have been reported: antiobesity, antioxidant, antiangiogenic, anti-inflammatory, anticancer, antibacterial, neuroprotective, photoprotective, antiviral, antiosteoporotic, regulating intestinal microflora, antiparasitic, hepatoprotective and eye protective [118,119]. Consequently, this carotenoid may be viewed as a promising multifunctional nutrient. In this view, due to its low bioavailability and solubility, many studies are trying to improve its absorption and solubility using various delivery and emulsifying products [119].

As fucoxanthin bioavailability is concerned, various evidence suggests that, after oral ingestion in animals, the carotenoid is converted to fucoxanthinol (see Figure 2 for the chemical structure) in the gastrointestinal tract and then metabolized to amarouciaxanthin A in the liver [120,121]. Fucoxanthin may be found at small concentrations in plasma and other tissues only after prolonged daily administration, i.e., at least a week [120]. Larger absorption of fucoxanthin may be obtained if administered along with edible oil or lipid [122,123], while it is only poorly adsorbed when ingested as a component of seaweed [124]. A further interesting aspect of use of fucoxanthin as a nutrient in the market is its lack of toxicity. Many studies have investigated the toxicity by fucoxanthin and fucoxanthinol, and, in a 13-week sub-chronic study with orally administered fucoxanthin, a NOAEL (No Observed Adverse Effect Level) of 200 mg/kg was reported [125]. Moreover, no mutagenicity was observed with the carotenoid [126,127]. A recommendation of EFSA for fucoxanthin extracted from thalli of the macroalga *Undaria pinnatifida* indicated a safe dose of 15 mg/day, and, in a recent pilot clinical trial, fucoxanthin was observed to be safe up to 30 mg/day [128]. Adverse effects of the marine carotenoid differently conjugated for better bioavailability were also investigated. No adverse effect of acute and sub-acute toxicity in rats of chitosan nanogels (CS-NGs) loaded with fucoxanthin with glycolipids was reported in the paper of Ravi et al. (2015). Moreover, Lykov et al. [129] demonstrated no toxicity of a composition of fucoxanthin with particles of porous aluminum oxide with polydimethylsiloxane on mice splenocytes and thymocytes, both under short-term (24 h) and long-term (129 h) incubation. For more information on the pharmaco/toxicological profile of fucoxanthin and its delivery strategies, several recent reviews are available [119,130,131,132,133].

Only a few studies evaluating the activities of fucoxanthin or other carotenoids extracted from diatoms have been reported (Table 1) and have been published in recent years. Neumann and collaborators [59], while studying fucoxanthin extracted from pennate diatom *P. tricornutum* (which is particularly rich in fucoxanthin), detected antiproliferative and antioxidant activity by this carotenoid. In contrast, they did not obtain evidence of an anti-inflammatory effect, studying the NO (nitric oxide) and cytokine release from RAW264.7 macrophage cells under lipopolysaccharide (LPS) stimulation. An effect on the LPS-induced inflammatory stimulus was, on the contrary, described for the main metabolite of fucoxanthin, fucoxanthinol (Figure 1), extracted from fucoxanthinol-rich diatom *N.a laevis* [134]. The pretreatment of BV-2 microglial cells with the carotenoid [134] significantly reduced expression of LPS-induced cyclooxygenase-2 (COX-2), release of tumor necrosis factor-α (TNF-α), interleukin-6 (IL-6), prostaglandin E2 (PGE-2) and nitric oxide (NO) and reactive oxygen species (ROS) production. These effects, overall, suggest potential neuroprotective activity promoted by the molecule. Recently, anti-inflammatory evidence was obtained also for fucoxanthin from *P. tricornutum* [63]. In this study, an inhibitory effect on NF-κB and NLRP3 inflammasome activation induced by the combination of LPS and ATP in murine-bone-marrow-derived macrophages and dendritic cells and in astrocytes was described.

### 3.2. Polyphenols

Polyphenols are secondary metabolites of plants, microalgae and macroalgae. They include more than 50,000 compounds. Usually, polyphenols are classified into ten different classes: hydroxybenzoic acids, hydroxycinnamic acids, flavonoids, stilbenes, lignans, flavones, isoflavones, flavanones, anthocyanidins and tannins [6]. The phenolic compounds are mainly structural components of cell walls in spermatophytes and algae, and most of them are involved in defense processes, coloring and antioxidant activities. During growth of superior plants, microalgae and macroalgae, the secondary metabolism produces a large and diverse class of polyphenols to protect organisms from environmental stresses, such as UV irradiation and pathogens [6,57]. In detail, phenolic compounds act directly against radical species as well as indirectly via inhibition of pro-oxidant enzymes, such as lipoxygenase, or through metal chelation, preventing occurrence of Haber–Weiss and Fenton reactions, which are important sources of radical species [135]. Indeed, polyphenols are the most widespread antioxidant substances in photosynthetic organisms [136]. Regarding disease and nutrition, thousands of papers have explored the biological activities of polyphenols, such as antioxidant, anti-inflammatory, skin-protective, antimicrobial, promoting apoptosis of tumor cells, estrogen-like action and neuroprotective effects [6,57]. In recent years, polyphenols have been identified in such diatoms as *N.a laevis* [74], *N. palea* [40], *P. tricornutum* [75] and *S. marinoi* [76].

Extracts derived from the culture of well-studied diatom *P. tricornutum* revealed the presence of 14 phenolic compounds, i.e., gallic acid, protocatechuic acid, catechin (see Figure 2 for the chemical structures), vanillic acid, epicatechin, syringic acid, chlorogenic acid, gentisic acid, caffeic acid, coumaric acid, ferulic acid, rutin, myricetin and quercetin [75]. Rico and collaborators [13] highlighted that increasing the levels of copper added to the culture media resulted in a general increase in phenolic contents in *P. tricornutum* cells, suggesting involvement of polyphenols in protection against metal toxicity. Furthermore, the authors demonstrated antioxidant efficiency of the diatom extracts against the stable radical 1,1-diphenyl-2-picrylhydrazyl (DPPH) [75], and the same results were reported by evaluating three fractions (hexane fraction, ethyl acetate fraction and water fraction) from *N. laevis* [74]. Smerilli and collaborators [14] reported results showing *S. marinoi* becoming rich in phenolic compounds under light manipulation [76]. Accordingly, at midday, *S. marinoi* increased production of antioxidants, counteracting the detrimental effect of reactive oxygen species (ROS), which are produced as a consequence of light exposure. Among the phenolic compounds produced by *S. marinoi* there are flavonoids. Flavonoids are located in different organelles, including chloroplasts, where they play a key photoprotective role; in particular, they can scavenge radical species and stabilize cell membranes [137]. This was the first study underlining the photoprotective role of polyphenols in diatoms.

Moreover, extracts of another diatom, *Nitzschia palea*, showed antioxidant properties in eight assays tested (DPPH, ferrous ion chelating, nitric oxide scavenging, ABTS radical scavenging, deoxy-ribose assay, superoxide radical scavenging activity (Nitroblue tetrazolium method), lipid peroxidation and ferric reducing antioxidant power (FRAP)) [40]. Interestingly, Lakshmegowda and colleagues showed the capability of the extract of *Nitzschia palea* to prevent DNA and protein damage, as well as anti-hemolytic properties comparable with standard synthetic antioxidants. Moreover, they tested the extract on in vitro murine macrophage (RAW 264.7) cell line and an in vivo mouse model. The extract of the diatom *Nitzschia palea* inhibited the protein expression of inflammatory cytokines, such as NO, TNF-α, IL-6 and PGE_2_. In in vivo experiments, the mice were treated with the extract of *N. palea* 100 or 200 mg/kg of body weight by oral route for 7 days. To induce paw edema, 1% carrageenan solution in physiological saline was injected at the sub-plantar region of the left hind paw and the volume of the paw edema was measured after 4 h in order to evaluate the power of the extract to prevent edema. Results of in vivo studies showed paw edema inhibition in extract-fed mouse group in line with the inhibition of COX-2 and myeloperoxidase (MPO) activity in the paw tissues [40].

## 4. Innovative Cultivation System and Extraction Methods to Meet the Challenging Demands of Nutraceutical Industries

### 4.1. Culture Systems

The chemical composition of microalgae is greatly variable according to key environmental factors [25,138], such as water temperature [139], salinity [140], light [141] and nutrient availability [25]. In addition, the production technologies applied largely influence production of bioactive compounds [24], while, in outdoor conditions, the environmental influences cannot be controlled because they vary with the seasons [142]. In closed conditions (e.g., cultivation in photobioreactor systems), production is triggered by perfectly controlled conditions [143]. However, outdoor cultivation is normally conducted in extensive conditions and, consequently, is economically more convenient for mass production [84]. In outdoor systems, massive production of various microalgae, including planktonic diatoms, may be obtained using both raceways and ponds. Open ponds and raceways are generally conducted as extensive means, intended to be low-technology and low-effort productive systems. This makes them sufficiently productive and economically feasible, which explains why they still account for about 99% of the world’s total production so far. Nevertheless, algal cultures have primarily been obtained for biofuel production or feeding purposes in recent decades, and the specific content in secondary metabolites could not represent an issue [27]. Newer applications for biotechnological applications (nutraceuticals, pharmaceuticals, etc.) impose obtaining consistent compositions to maximize production of selected metabolites [144]. To this end, it is likely that indoor production in photobioreactors, assuring stability of culture productions and the possibility to modulate environmental conditions, will drastically increase in the future [47].

### 4.2. Methods of Extraction of the Active Compounds

Aside from the methods of production, the methods of extraction highly influence composition of diatom extracts. The choice of an appropriate method of extraction is critical to obtain sufficient amounts of the active biomolecules, both for analytical and biotechnological purposes [48]. In fact, diatoms produce both constitutive and wound-activated compounds [49] through some specific metabolic pathways. Constitutively produced compounds may be excreted as hydrosoluble molecules (e.g., the pigment “marennin” constitutively excreted by *Haslea ostrearia*; [47]) or emitted through the pores on the thecae and linked to polysaccharides used for attachment to the substratum, in the case of benthic species or in planktonic diatoms producing masses of gelatinous aggregates [50]. In this case, production of exudates may vary during diatom cell cycles and mainly occurs during the declining growth phase and continues during the stationary phase or, in culture, when cell division, growth and photosynthesis are unbalanced. Wound-activated compounds are often produced through the lipoxygenase pathway [51] and are represented by both volatile organic compounds (VOCs) as oxylipins and non-volatile compounds as FAs. Plant oxylipins are a group of “signaling molecules”, and the same diatom biosynthetic pathway also leads to oxygenated FAs, aldehydes and other metabolites often used as grazing deterrents [33]. In fact, oxylipins are oxygenated derivatives of PUFAs, representing chemical mediators in several ecological and physiological processes in both marine and freshwater diatoms. Their biosynthetic pathway includes VOCs with known info-chemical purposes [49] and also small FAs with extraordinary bioactivity [37], tuning and triggering various metabolic functions [52]. The main challenge of culture techniques and extraction methods is represented by the large variability in the metabolism of a given species and the plethora of compounds that may be present in extracts, eventually interacting during extraction and fractionation processes [25,91,116].

The most common systems of extraction involve complete destruction of diatom thecae by means of mechanical (pottering) or ultrasound methods and further homogenization of the cell’s content. Further, the homogenized content is extracted using organic solvents or mixtures of polar and apolar solvents. Kato et al. [48] suggest to apply high-pressure extraction under a nitrogen atmosphere using hydrophobic solvents, adopting a mixture of chloroform and ethyl-acetate. This method permits to obtain a full set of FAs of biotechnological interest. However, diatoms also contain polar or partially polar compounds that have interesting bioactivity, such as the domoic acid that is often produced as a deterrent. Nijjar et al. [53] proposed a method of extraction and purification of partially polar compounds from diatoms using a mixture of chloroform and methanol (1:2, *v*/*v*), followed by ultrafiltration through a PM1 Millipore filter and repeated high-performance liquid chromatography on a reversed-phase column. This permitted improved recovery with comparable purity of domoic acid. Another method often applied is complete extraction with pure methanol [54], or with a mixture of methanol and dichloromethane (1:2), adopting a “Folch-like” lipid solvent followed by a partially polar solvent (20% ethanol and 80% ethanol-diethyl ether) used in a sequential extraction method. This method permitted to collect most organic substances in the two sequential fractions.

Other investigations aimed at complete extraction of pigments and other polar organic substances, for which hard hydrophobic solvents are not appropriate. To this end, Hagerthey et al. [145] proposed a simple one-stage extraction protocol and compared the performances of four solvents (acetone, methanol, methanol/acetone and methanol/acetone/N, *N*-dimethylformamide), also considering the effect of grinding vs. freeze-drying as homogenization methods. They demonstrated that the method based on methanol/acetone/DMF/water (MAD) was the most effective to extract polar organic compounds as pigments.

In contrast, extraction of highly polar compounds derived from the crucial lipoxygenase pathway requires mostly polar solvents to obtain high yields. Cutignano et al. [146] proposed a methodology based on selective enrichment of the oxylipin fraction by methanol extraction, followed by parallel acquisition of full-scan UV and tandem mass spectra on reverse phase liquid chromatography (LC) peaks. Their method enables easy identification of genetic differences, enzymatic regulation and environmental conditions triggering variations in oxylipin signatures. However, VOC compounds and highly lipophilic metabolites may be underestimated using this method. In addition, synthesis of wound-activated compounds is likely to be scarce when the solvent is directly added to the homogenates because the necessary biosynthetic reactions may be prevented out of the aqueous environment [147].

To this end, Zupo et al. [91] proposed a more efficient method based on such a highly apolar solvent as acetonitrile. In particular, freeze-dried biomass of diatoms, after 10 min of activation in distilled water, was extracted with aqueous 80% acetonitrile. The soluble part was separated from the insoluble part by centrifugation (10 min) in Corex glass tubes at 7400× *g* (DuPont Instruments, Newtown, CT, USA). The extract was further separated into five fractions on an analytical C18 reversed phase HPLC column (GromSil ODS 4 HE, 250 × 4.6 mm, 5 μm particle size; Stagroma, Germany; 1 mL/min flow rate; 30 °C oven temperature; diode array detection) using a linear gradient of solvent A (80/20, *v*/*v*, MeOH/H_2_O) and solvent B (80/20, *v*/*v*, MeOH/acetone). This double-stage separation facilitated full recovery of a higher amount and variety of FA s and VOCs as compared with other extraction and fractionation methods.

## 5. Conclusions and Future Perspectives and Challenges of Diatoms as Nutraceuticals

Diatoms represent, from a nutraceutical point of view, valuable marine organisms thanks to their content in molecules with a recognized role for human health. The compounds currently considered as the most interesting for direct extraction from diatoms are carotenoid fucoxanthin, polysaccharide chrysolaminarin and omega-3 PUFAs EPA and DHA. Some experiments also report interesting levels and activities of proteins, peptides and polyphenols. In contrast, the data reported about the activities of diatom-derived sterols and on levels of minerals and vitamins are still insufficient.

**Figure 3:**
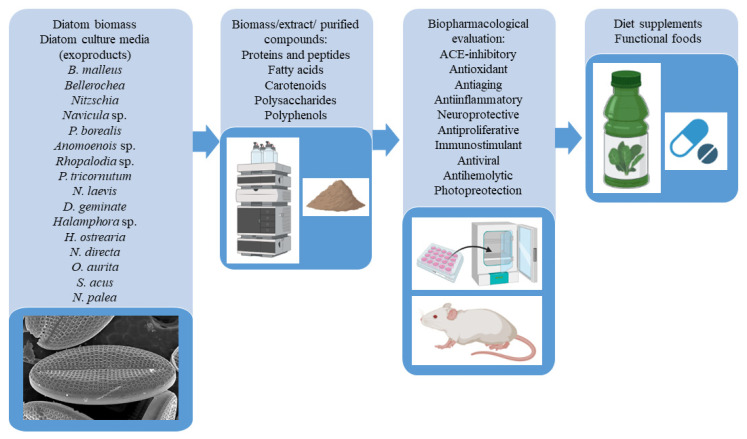
Pipeline adopted to obtain potential use of diatoms as biomass, extracts or purified compounds in functional food and diet supplements.

High-value compounds or analogues (laminarin for chrysolaminarin) have been widely studied for their health benefits as pure compounds purified mostly from other organisms. Currently, only 23 studies had a focus on the activities of compounds directly extracted from diatoms and little evidence has been published on use of diatoms in the form of biomass at preclinical and clinical levels. Consequently, the bio-pharmacological investigations of diatoms need to be improved in order to exploit the relevant role of these microalgae for the nutraceutical industry (Figure 3).

*P. tricornutum* is currently the most studied and characterized diatom and is the source of an EPA-rich oil already used as a supplement in the USA, approved by the FDA, while currently under evaluation in Europe by the EFSA. This diatom represents a good example of how, in the last decade, application of molecular biology techniques in the biotechnological field, as well as the nutraceutical one, is driven by the rapidly decreasing cost and increasing throughput of DNA-sequencing in meeting the urgent need to discover new drug entities to counteract the increased incidence of severe diseases and the reduced efficacy of existing drugs. In fact, the availability of the *P. tricornutum* genome sequence has helped in reconstruction of its metabolic network, which, in turn, makes it possible to predict the metabolic capabilities of this diatom from its genotype, performing in silico knock-ins of heterologous pathways and knockouts. Systems biology approaches are very promising in engineering of diatoms for value-added products. Furthermore, in the genomic era, when the number of available genomes is increasing, genome mining is offering a significant push in identification of previously uncharacterized natural products and their biosynthetic gene clusters, joined to sequence analysis of the enzymes encoded by these gene clusters, complementing chemical identification of the products of gene clusters. Genome mining obtains strong support from synthetic biology, which permits to design and then construct new biological enzymes and genetic circuits and/or redesign of existing biological systems. It is noteworthy that diatoms exhibit all the advantages of photosynthetic eukaryotic systems but lack many of the disadvantages of superior plant-based expression systems. In fact, diatoms are easy to grow, with high growth rates, but they do not require an external source of organic carbon for cultivation.

Another diatom success on the nutraceutical market is that of *O. aurita*, whose biomass received a positive opinion from the EFSA regarding its use as a food ingredient. Several other diatoms have been studied for their content and potential as dietary supplements or ingredients in functional foods, looking mostly at their content of FAs, carotenoids and polysaccharides. Surely, in the near future, some of them will reach the nutraceutical market, but only their evaluation through preclinical studies and clinical trials, will provide indispensable evidence for correct use for human health.

It is worth observing, as initially highlighted, that investigations on the nutraceutical value of diatoms are mostly limited to a few species of marine planktonic diatoms. This evident limitation, due to a historical attitude of scientific research, represents a remarkable constraint because of the wide diversity of biosynthetic pathways characterizing various families of diatoms and the evolutionary triggers that elicited development of various functional compounds according to the habitats. Consequently, the main challenge for the future is represented by the need to widen our research perspectives, including more species of benthic diatoms and taking into account the peculiarities of freshwater species. This effort will be sufficient to boost discovery of new compounds and new activities for formulation of modern functional foods and for several nutraceutical applications. In this view, the results herein reported represent only the start of a long research story that is destined to develop in subsequent decades.

## Data Availability

Not applicable.
